# *Gwakhyangjeonggi-san* for irritable bowel syndrome: A protocol for systematic review and meta-analysis

**DOI:** 10.1097/MD.0000000000026635

**Published:** 2021-07-09

**Authors:** Jongwon Park, Seok-Jae Ko, Gajin Han, Keumji Kim, Hyejin Jun, Jae-Woo Park

**Affiliations:** a Department of Clinical Korean Medicine, Graduate School of Kyung Hee University, Seoul, Republic of Korea; b Department of Gastroenterology, College of Korean Medicine, Kyung Hee University, Seoul, Republic of Korea; c JINRESEARCH, Seoul, Republic of Korea; d Sweet & Sunny Korean Medicine Clinic, Seoul, Republic of Korea; e Department of Internal Medicine, Kyung Hee University Hospital, Gangdong, Seoul, Republic of Korea.

**Keywords:** *Gwakhyangjeonggi-san*, irritable bowel syndrome, randomized controlled trial, systematic review

## Abstract

**Background:**

Irritable bowel syndrome (IBS) is a chronic functional bowel disorder characterized by abdominal pain or discomfort, stool irregularities, and bloating. Owing to its atypical symptoms and various mechanisms, there is no standard treatment for IBS. *Gwakhyangjeonggi-san* (GJS), a traditional Korean herbal medicine, has been used to treat lower intestinal abnormalities in Asia. We will systematically review randomized controlled trials (RCTs) to evaluate the efficacy and safety of GJS as a complementary treatment for IBS.

**Methods and analysis:**

Four English databases, namely, Medline (via PubMed), EMBASE, the Cochrane Central Register of Controlled Trials, and the Allied and Complementary Medicine Database, will be searched for entries up to May, 2021. Additional databases will include 5 Korean databases, 1 Chinese database, and 1 Japanese database. RCTs and quasi-RCTs will be searched for to assess the effectiveness and safety of GJS. The primary outcome measure will be the overall efficacy rate, and the secondary outcome will include data such as global symptom scores, IBS Quality of Life measurements, and adverse events. Data analysis will be performed using Review Manager Version 5.3, and the risk of bias will be assessed using the Cochrane Collaboration's risk-of-bias tool. The quality of the results will be evaluated using the Grading of Recommendations Assessment, Development, and Evaluation approach.

**Conclusion:**

This systematic review will provide evidence for the efficacy and safety of GJS for IBS.

**OSF registration number:**

DOI 10.17605/OSF.IO/V93JN (https://osf.io/v93jn).

## 1 Introduction

Irritable bowel syndrome (IBS) is a functional bowel disorder characterized by abdominal pain or discomfort, stool irregularities, and bloating without structural or biochemical abnormalities that can be detected by current routine diagnostic tools.^[1]^ IBS is also known as a chronic disease of a cyclic nature characterized by recurrent symptoms.^[2]^ According to a meta-analysis of the prevalence of IBS published in 2012, the global prevalence was 11.2%.^[3]^ IBS is known to be associated with various mechanisms such as abnormal motility, visceral hypersensitivity, enhanced pain perception, dietary intolerance, abnormalities in the autonomic nervous system, biological and psychosocial stressors, and bidirectional communication between the neural and immunological networks.^[4]^ Conventional treatment for IBS, including fiber, antidiarrheal agents, smooth muscle relaxants, and psychotropic agents, has been unsatisfactory due to limitations such as lower effectiveness with long-term use, exacerbation of abdominal pain induced by anticholinesterase neostigmine, and poor compliance with psychotropic agents.^[5]^ The need for complementary and alternative medicines to treat IBS has arisen.^[6]^

Herbal medicine has been used in Asia in place of Western medicine owing to its safety and fewer side effects.^[7]^*Gwakhyangjeonggi-san* (GJS), a Korean herbal medicine (*Kkako-shoki-san* in Kampo Medicine and *Huoxiang-zhengqi-san* in Traditional Chinese Medicine), has been used to treat symptoms of lower intestinal abnormalities such as diarrhea and lower abdominal pain.^[8,9]^ GJS consists of *Agastache rugosa* (5.63 g), *Perilla frutescens* (3.75 g), *Angelica dahurica* (1.88 g), *Areca catechu* (1.88 g), *Poria cocos* (1.88 g), *Magnolia officinalis* (1.88 g), *Atractylodes macrocephala* (1.88 g), *Citrus unshiu* (1.88 g), *Pinellia ternata* (1.88 g), *Platycodon grandiflorum* (1.88 g), *Glycyrrhiza uralensis* (1.88 g), *Zingiber officinale* (3.75 g), and *Ziziphus Jujuba* (3.75 g). Despite its wide usage, there is a lack of research on the clinical efficacy and safety of GJS.^[10]^

In the proposed study, we will systematically review and meta-analyze the efficacy and safety of GJS for IBS. The study will provide evidence for the applicability of GJS as complementary medicine for the treatment of IBS.

## 2 Methods

### 2.1 Study registration

A protocol has been registered on OSF (https://osf.io/v93jn).

### 2.2 Inclusion criteria for study selection

#### 2.2.1 Types of studies

The systematic review will include randomized controlled trials (RCTs) and quasi-RCTs. Animal research, case studies, and commentaries will be excluded.

#### 2.2.2 Types of patients

Patients who meet the ROME diagnostic criteria irrespective of age, gender, or race will be included in the study. As the ROME criteria standard was first announced in 1992 and the latest version of the criteria (ROME IV) was released in 2016,^[11]^ studies published before 1992 will be screened for eligibility, and criteria similar to the ROME criteria (eg, Manning and Kruis criteria)^[12]^ will be included under an agreement between researchers. Studies in which IBS patients were diagnosed with other organic intestinal diseases, such as ulcerative colitis, Crohn disease, or colorectal cancer, will be excluded.

#### 2.2.3 Types of interventions

Studies using GJS and herb-added GJS (modified GJS) for IBS will be included. The comparator groups will be groups taking conventional Western medicines and placebos of the same color, appearance, and odor as GJS, and the waiting group without treatment. Western medication interventions include antidiarrheal agents, smooth muscle relaxants, and psychotropic agents.

#### 2.2.4 Types of outcome measures

The primary outcome will be the overall efficacy rate. The secondary outcome will be data such as global symptom scores, IBS Quality of Life measurements, and adverse events.^[13,14]^

### 2.3 Data source and data collection procedures

#### 2.3.1 Database for searching

We will search the following 11 electronic databases for entries up to May, 2021 without language or publication date restrictions. Four English databases, namely, Medline (via PubMed), EMBASE, the Cochrane Central Register of Controlled Trials, and the Allied and Complementary Medicine Database, will be searched. Five Korean databases, namely, the Korean Studies Information Service System, National Digital Science Library, Korean Medical Database, KoreaMed, and Oriental Medicine Advanced Searching Integrated System , will also be included. One Chinese database, China National Knowledge Infrastructure, and 1 Japanese database, Citation Information by NII, will be searched. The search terms will be composed independently or in conjunction with the disease and intervention. The search strategy for Medline via PubMed is presented in Table 1.

**Table 1:** Search terms for Medline via PubMed.

#1.	Irritable Bowel Syndrome[MH] OR “Irritable Bowel Syndrome” OR “irritable Bowel Syndromes” OR “Syndrome, Irritable Bowel” OR “Syndromes, Irritable Bowel”
#2.	“Colon, Irritable” OR “Irritable Colon”
#3.	“Colitis, Mucous” OR “Colitides, Mucous” OR “Mucous Colitides” OR “Mucous Colitis”
#4.	“Colonic disease, functional” OR “Irritable Bowel” OR “Spastic colon” OR “functional bowel disease” OR “functional colonic disease” OR Colonic Diseases, Functional[mh]
#5.	“irritable bowel syndrome”[tw] OR irritable bowel syndrome^∗^[tw] OR IBS[tw] OR “functional abdominal dpain”[tw] OR “functional gastrointestinal disorders”’[tw]
#6.	#1 OR #2 OR #3 OR #4 OR #5
#7.	*Gwakhyangjeonggi-san* OR *Gwakhyangjeonggisan* OR “*Gwakhyangjeonggi san”*
#8.	“*Huo Xiang Zheng Qi San”* OR “*HuoxiangZhengqi San”* OR “*Huo Xiang Zheng Qi Tang”* OR *Huoxiangzhengqisan*
#9.	*Gamigwakhyangjeonggi-san* OR *GamiGwakhyangjeonggisan* OR “*GamiGwakhyangjeonggi san”*
#10.	“*Jiawei Huo Xiang Zheng Qi San”* OR “*Jiaweihuoxiangzhengqisan”* OR “*Jiawei-Huo-Xiang-Zheng-Qi-San”* OR “*Jiawei HuoXiangZhengQi San”*
#11.	*kakkoshokisan* OR *Kamikakkoshokisan* OR *kakkousyoukisan* OR *Kamikakkousyoukisan*
#12.	#7 OR #8 OR #9 OR #10 OR #11
#13.	#6 AND #12

IBS = irritable bowel syndrome.

#### 2.3.2 Data selection and exclusion

**Figure 1:**
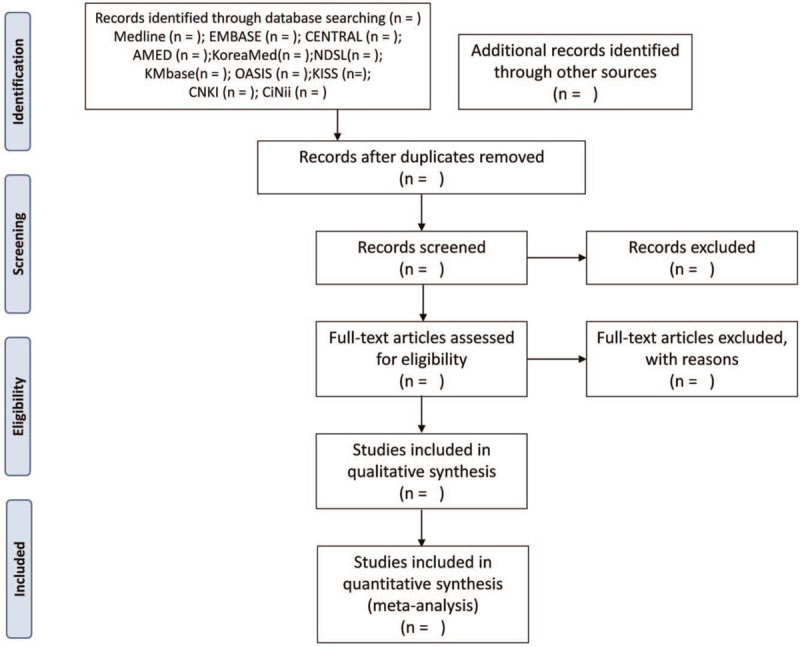
PRISMA flow diagram of literature screening and selection process. AMED = Allied and Complementary Medicine Database, CENTRAL = Cochrane Central Register of Controlled Trials, CiNii = Citation Information by Nii, CNKI = China National Knowledge Infrastructure, KISS = Korean studies Information Service System, KMbase = Korean Medical Database, NDSL = National Digital Science Library, OASIS = Oriental Medicine Advanced Searching Integrated System, PRISMA = Preferred Reporting Items for Systematic Reviews and Meta-Analyses.

Two independent researchers (SJK and GH) will sequentially screen the titles, abstracts, and main texts to select studies that meet the research purpose. The final selected studies will be determined through an agreement between the 2 researchers, and if they fail to reach an agreement, a third party (JWP) will intervene. Endnote X7 (Clarivate Analytics, London, UK) will be used for study screening. This process is shown in the Preferred Reporting Items for Systematic Reviews and Meta-Analyses diagram (Fig. 1).

#### 2.3.3 Data extraction

Data extraction will proceed in a predefined format according to the “Consolidated Standards of Reporting Trials Extension for Chinese Herbal Medicine Formulas 2017.” It consists of research information (title, abstract, and keywords), introduction (background and objectives), method (trial design, participants, intervention, outcomes, sample size, randomization sequence generation, and allocation concealment mechanism), results (participant flow, recruitment, and baseline data), discussion (limitation, generalizability, and interpretation), and other information (registration, protocol, and funding).^[15]^ Missing or insufficient data in the study will be filled in by placing requests with the corresponding authors of the studies.

### 2.4 Quality assessment

The Cochrane Collaboration's risk-of-bias tool will be used to assess the risk of bias. The Cochrane Collaboration's risk-of-bias tool evaluates the bias in the domains of random sequence generation, allocation concealment, blinding of participants and personnel, blinding of outcome assessors, completeness of outcome data, selective reporting, and other biases. Each domain will be judged as low risk, uncertain risk, or high risk.^[16]^

We will also use the Grading of Recommendations Assessment, Development, and Evaluation approach to assess the quality of evidence. Two researchers will evaluate each domain, which consists of downgrading factors such as the risk of bias, inconsistency, indirectness, imprecision of the results, and publication bias, and upgrading factors such as large effect and dose-dependent response.^[17]^

### 2.5 Data analysis and synthesis

#### 2.5.1 Analysis and synthesis strategy

We will quantitatively synthesize the extracted data using Review Manager Version 5.3 software (Cochrane Collaboration, Oxford, UK). The dichotomous outcome will be represented as a risk ratio with a 95% confidence interval (CI), whereas the continuous outcome will be represented as a mean difference or standardized mean difference with 95% CI. Statistical heterogeneity among studies will be assessed using the *I*^2^ statistic, and when the studies show significant heterogeneity (*I*^2^ ≥ 50%), the random-effects model will be applied. Sensitivity analysis will be carried out to enhance the robustness of the results. Funnel plots will be generated to assess the publication bias in cases where the number of sources is more than 10.

#### 2.5.2 Analysis of subgroups or subsets

Subgroup analysis will be conducted according to the type of Western medication, dose of intervention, treatment duration, or subtype of IBS (diarrhea- or constipation-predominant IBS).

### 2.6 Ethics and dissemination

As the study only involves a protocol for a systematic review, no ethical approval is required. The results of the study will be published in peer-reviewed journals and distributed electronically or in print.

## 3 Discussion

IBS lowers not only the quality of life of an individual but also their productivity, owing to its chronic characteristics and the absence of standard therapy. According to a long-term follow-up study, more than 50% of patients with IBS have symptoms 7 years later.^[18]^ A systematic review published in 2013 reported that the direct costs for IBS treatment per patient ranged from $1562 to $7547, and the indirect costs ranged from $791 to $7737 per year in the United States.^[19,20]^

Although GJS has long been used to treat lower intestinal abnormalities such as diarrhea and abdominal pain,^[8,9]^ its efficacy and safety have not been sufficiently investigated. A recent study on the effect of GJS on the intestinal microbiota of IBS patients showed that the proportion of beneficial bacteria increased when GJS and probiotics were administered simultaneously.^[7]^ Through a systematic review, we will investigate the synergistic effect of GJS and conventional Western medicine or probiotics.

The study will provide evidence on the efficacy and safety of GJS as a complementary treatment for IBS, and the findings will help IBS patients, healthcare providers, and health policymakers in decision-making.

## Author contributions

**Conceptualization**: Seok-Jae Ko, Jae-Woo Park.

**Funding acquisition:** Jae-Woo Park.

**Methodology:** Jongwon Park, Seok-Jae Ko, Gajin Han, Keumji Kim, Hyejin Jun.

**Supervision:** Seok-Jae Ko, Jae-Woo Park.

**Writing – original draft:** Jongwon Park.

**Writing – review & editing:** Seok-Jae Ko, Jae-Woo Park.
